# MetaPath: identifying differentially abundant metabolic pathways in metagenomic datasets

**DOI:** 10.1186/1753-6561-5-S2-S9

**Published:** 2011-05-28

**Authors:** Bo Liu, Mihai Pop

**Affiliations:** 1 Center for Bioinformatics and Computational Biology, Institute for Advanced Computer Studies, University of Maryland, College Park, MD 20742, USA; 2 Department of Computer Science, University of Maryland-College Park, College Park, MD 20742, USA

## Abstract

**Background:**

Enabled by rapid advances in sequencing technology, metagenomic studies aim to characterize entire communities of microbes bypassing the need for culturing individual bacterial members. One major goal of metagenomic studies is to identify specific functional adaptations of microbial communities to their habitats. The functional profile and the abundances for a sample can be estimated by mapping metagenomic sequences to the global metabolic network consisting of thousands of molecular reactions. Here we describe a powerful analytical method (MetaPath) that can identify differentially abundant pathways in metagenomic datasets, relying on a combination of metagenomic sequence data and prior metabolic pathway knowledge.

**Methods:**

First, we introduce a scoring function for an arbitrary subnetwork and find the max-weight subnetwork in the global network by a greedy search algorithm. Then we compute two *p* values (*p_abund_* and *p_struct_*) using nonparametric approaches to answer two different statistical questions: (1) is this subnetwork differentically abundant? (2) What is the probability of finding such good subnetworks by chance given the data and network structure? Finally, significant metabolic subnetworks are discovered based on these two *p* values.

**Results:**

In order to validate our methods, we have designed a simulated metabolic pathways dataset and show that MetaPath outperforms other commonly used approaches. We also demonstrate the power of our methods in analyzing two publicly available metagenomic datasets, and show that the subnetworks identified by MetaPath provide valuable insights into the biological activities of the microbiome.

**Conclusions:**

We have introduced a statistical method for finding significant metabolic subnetworks from metagenomic datasets. Compared with previous methods, results from MetaPath are more robust against noise in the data, and have significantly higher sensitivity and specificity (when tested on simulated datasets). When applied to two publicly available metagenomic datasets, the output of MetaPath is consistent with previous observations and also provides several new insights into the metabolic activity of the gut microbiome. The software is freely available at http://metapath.cbcb.umd.edu.

## Background

Metagenomics is a new scientific field that involves the analysis of organismal DNA sequences obtained directly from an environmental sample, enabling studies of microorganisms that are not easily cultured in a laboratory [1]. Metagenomic studies, pioneered in the early 2000s [2], have recently increased in number and scope due to the emergence of next generation sequencing technologies. Due to the difficulty of assembling entire organisms from a metagenomic dataset [1], most analyses take a gene-centric view, treating the community as an aggregate and ignoring the exact assignment of genes to individual organisms. In fact, it can be argued that the environment is better characterized by its gene complement rather than by its taxonomic composition, given that similar biological functions can be performed by microbes of distinct taxonomic origins. Supporting this view is the observation that, while the taxonomic composition of the human gut microbiome varies significantly between people, the functional profile is remarkably stable across samples [3]. The functional profile for a sample can be recovered by mapping sequences to gene families [4], subsystems [5] or metabolic pathways [6]. The relative abundance of each functional category can be estimated by counting how many sequences are assigned to each category, and this information is the basis for detailed comparisons of the functional potential of different functions. In a typical comparative metagenomics experiment, random shotgun sequences are generated from a collection of samples belonging to two groups, for example, obese or lean twins [3], and healthy infants or adults [7]. An important biological problem is to find differentially abundant functional signatures (e.g., genes or metabolic pathways) that are selected for by their local environments. Traditional analysis approaches compare the relative abundances of the categories one-at-a-time between different phenotypes, and compute the significance using one of several statistical approaches [8-10]. When comparing communities at the gene family level, many functional categories are commonly found to be differentially abundant, even after correcting for multiple hypothesis testing [3,7]. The interpretation of these data can be daunting. An alternative approach focuses on functional subsystems and metabolic pathway comparisons [11], the number of which is much smaller than gene families. Results at these levels are easier to interpret and can provide a stronger evidence of distinct functional capacities than at the level of individual gene families. Such analyses, however, can be unnecessarily coarse. For example, the use of KEGG pathways as a basis for analysis is complicated by the following issues: (1) the definitions of pathways in KEGG are coercive, and the interactions between these pathways are ignored; (2) the genes in a pathway may not be fully covered by the identified genes in a metagenomic sample; (3) significant differences in the abundance of certain genes may be masked once the abundance of all genes in a pathway is aggregated.

To address these problems, we introduce a general method (MetaPath) for searching the global metabolic network to find differentially abundant finer-level subnetworks. For the purposes of this paper we define a subnetwork to be a connected set of genes that is statistically enriched or depleted in one group of samples. Underlying our approach is a statistical scoring system that captures the differential abundance for a given subnetwork, combined with a greedy search algorithm for a maximum weighted subgraph, to indentify the highest scoring subnetworks. Unlike previous approaches, MetaPath explicitly searches significant subnetwork in the global metabolic network (rather than the KEGG defined pathways), enabling us to detect subnetworks spanning predefined “containers”. In addition, we developed rigorous statistical methods that take into account the topology of the network when testing the significance of the subnetworks.

Using simulated datasets, we demonstrate that Metapath outperforms previously described approaches for comparing biological networks based on abundance data. We show that our findings are more robust to noisy data than the results of single gene comparisons, and that MetaPath can find finer-level subnetwork than can be found by comparing predefined KEGG pathways. We also discuss the biological significance of the results derived from the application of MetaPath to actual metagenomic datasets, demonstrating that the output from MetaPath is easy to interpret and provides valuable biological insights. The software is freely available at http://metapath.cbcb.umd.edu.

## Methods

### Datasets

**Figure 1:**
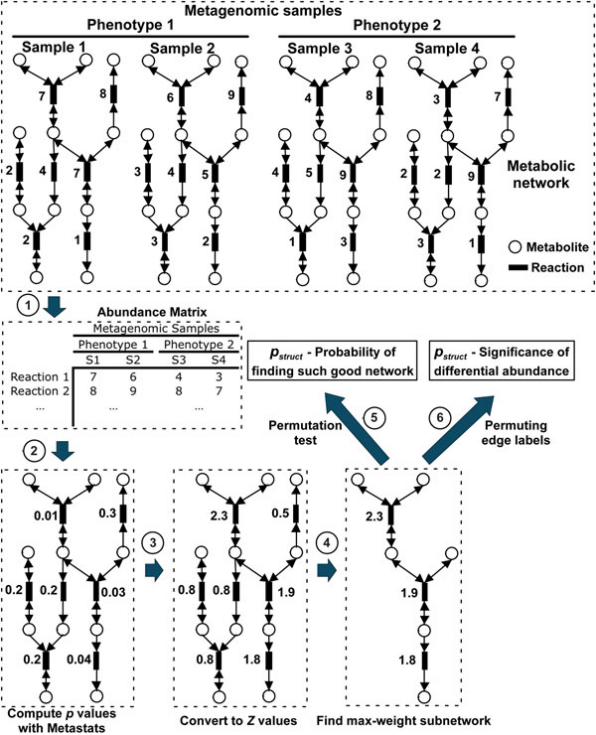
**Schematic diagram of the MetaPath methods**. Sequences from each sample are annotated against KEGG genes database and mapped to reactions in metabolic networks, resulting an abundance matrix where the rows are reactions and columns are samples. Then *p* values are computed for all reactions using Metastats [9], then converted into Z values, and greedy search is performed on the edge-weighted graph to find max-weight subnetworks. Finally, we calculate the p_abund_ and p_struct_ significance values of the max-weight subnetwork.

We tested our methods on two previously published metagenomic datasets, which were downloaded from the NCBI Trace Archive or Short Read Archive databases: (1) gut microbiomes from obese and lean twins [3]; (2) metagenomes from adult- and infant-type gut microbiomes [7]. Each dataset is divided into two populations of distinct phenotypes. The metabolic pathway data were downloaded from the KEGG pathways database [6]. The metabolic network is represented as a graph where nodes are metabolic substrates, and edges are molecular reactions (Fig. 1). The edges could be unidirectional or bidirectional depending on whether the corresponding reaction is reversible (as specified in KEGG database). Multiple reactions that are related to a same biological process are aggregated by KEGG into a “pathway” (e.g., glycolysis pathway). In addition, we refer to the network comprising all metabolic pathways in KEGG as the “global metabolic network”. Metagenomic sequences are annotated through BLASTX searches against KEGG genes database. The abundance of each molecular reaction is estimated as the number of metagenomic sequences mapped to it. Note that more accurate abundance estimates can be obtained by taking into account the length of individual genes [12] and we plan to explore the use of such estimates (and the associated statistics) in future versions of our software.

### Scoring metabolic subpathways

To score the biological activity of a particular subnetwork, we first use Metastats [9] to calculate the significance of differential abundance for each reaction between the two phenotypic groups under comparison. Under the null hypothesis, the relative abundances are randomly drawn from the same distribution across different phenotypic groups, thus the *p* value for each feature (metabolic reactions) follows a uniform distribution from 0 to 1. Based on this assumption, *p* values can be converted to *Z* scores [13] using the Gaussian distribution. Because Metastats performs a two-tailed test for each reaction, the two-tailed *p* values can be converted back to the original *Z* values using the following equation:

where  is the inverse cumulative density function (CDF) of standard normal distribution; G1 and G2 represent two different phenotypic groups. Using this formula, if a reaction is more abundant in population G1, then its *Z* score will be positive and vice versa. We are specifically interested in finding a pathway whose reactions are either enriched or depleted as a whole, as apposed to previous approaches [13,14] that identify active or perturbed subnetworks, which may contain a mixture of enriched and depleted components. Similar to the approach of [13] we define the aggregate score for a particular subnetwork to be the sum of the *Z* scores over all reactions contained within it: , where *k* is the size (number of metabolic reactions) of the subnetwork.

### Identifying high-scoring pathways

As proposed in [13], we attempt to find subnetworks that maximize the cumulative Z- score defined above. Unfortunately, this problem is NP-hard, which is equivalent to finding a maximum-weight subgraph [13]. Several approaches to solving this problem have been previously proposed: Ideker, *et al.* 2002 [13] used simulated annealing, but this heuristic is slow; Dittrich, *et al*, 2008 [14] used integer linear programming that can find provably optimal subpathways quickly, but it requires the commercial software package CPLEX that is not available to the general public (using a freely available ILP solver would require re-implementing the entire algorithm as the software is provided as a binary-only release). Here we rely on a greedy search heuristic that is fast, and, while not guaranteed to find maximally scoring pathways, performs well in practice. The algorithm we employ is described below:

This algorithm tries to find a connected metabolic subnetwork, which can have any arbitrary structure, with maximum weight. However, it is believed that in metabolic networks, chains are especially more biologically meaningful and interesting, because they attempt to capture the structure of a series of reactions that are successively connected. To allow this idea, we modify line 8 of the above algorithm to “Pick an edge e_j_ which has the highest weight of the edges that are adjacent to and have the same direction with e_j-1_”. Both searching algorithms are implemented in our program and can be selected through command-line parameters. To find all significant subnetworks (computing significance is discussed below), we iteratively remove the edges in the global network that are contained in previously found significant subnetworks, and rerun our greedy search on the rest of the network until we can no longer find any additional significant subnetworks. Note, that unlike the original version of our code [15], the search algorithm is not limited to given subnetwork size, rather will find all significant subnetworks irrespective of size.

### Computing the significance of subnetwork

**Figure 2:**
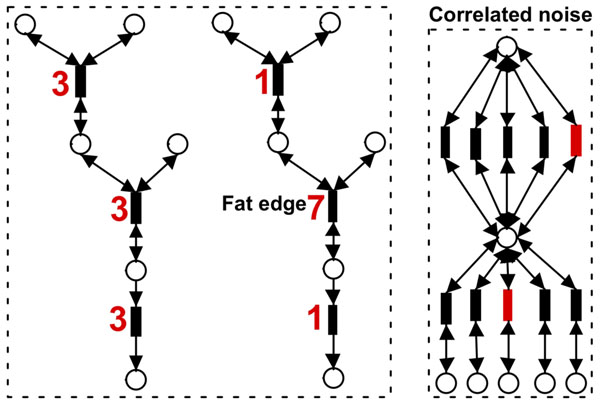
**Significant subnetworks that are caused by structural biases**. On the left side, both of the two pathways have equal weight, indicating equal significance of differential abundance. The high weight of the second pathway, however, mainly come from the middle fat edge that has weight 7. On the right side, in a densely connected network, any random high-weight edges will form a subnetwork with high weight (correlated noise).

The null score distribution for a specific subnetwork can be estimated by permuting the sample labels (columns of the abundance matrix) of the reactions and computing the subnetwork scores from the permuted abundance matrix. The significance *p* value is estimated as the number of random permutations that produce higher scores than the original subnetwork. The *p* value computed through this approach (termed *p_abund_* throughout the rest of the paper), however, ignores the topology of the underlying global metabolic network, and potentially leads to incorrect conclusions. For example, assume we have a densely connected metabolic network, in which every edge is connected with all other edges. Then, the best subnetwork is simply composed of the top differentially abundant metabolic reactions. This indicates that whenever there are significant reactions, which may simply come from random noise given the large number of edges, they will form a significant subnetwork because of the biases from the network topology (Fig. 2). To address this problem, we compute another *p* value (termed *p_struct_*), relying on a topological definition of the null distribution of subnetwork scores. Specifically, instead of treating each subnetwork as a bag of genes, we estimate the distribution of scores for actual subnetworks identified within the underlying global metabolic network. Since this null-distribution depends on the size (number of edges) of the subnetwork, let *k* be the size of a subnetwork generated by the greedy search algorithm described above, and *Z* be the corresponding *Z*-score. The *p_struct_* value for this subnetwork can be calculated as follows: (i) permute the edge weights (row labels of the abundance matrix) of the global metabolic network; (ii) perform greedy search to find a maximal weighted subnetwork of size *k*; (iii) repeat step 1 and step 2 for 1000 times, and generate 1000 weights of the max-weight subnetwork (null distribution); (iv) the *p_struct_* value is the proportion of the 1000 times in step 3 that we see scores higher than our original observation *Z*.

### MetaPath methods summary

To summarize the methods described above, the MetaPath algorithm proceeds as follows:

1. Differential abundance is assessed on an edge-by-edge basis (reaction-by-reaction) using Metastats;

2. The significance estimates (*p*-values) from Metastats are fed into a greedy search algorithm to determine all maximally weighted subnetworks(in terms of statistical *Z*-scores) in the global metabolic network;

3. The significance of each subnetwork detected by the greedy search algorithm is assessed using both a topology-independent bootstrapping approach (*p_abund_*), and a topology-dependent bootstrapping approach (*p_struct_*);

4. The subnetworks determined to be significant (*p_abund_* ≤ *0.05* and *p_struct_* ≤ *0.05*) are reported to the user (Note: the threshold for significance can be adjusted through command-line parameters). The pathways are ranked by *p_abund_* values.

## Results and discussions

### Performance evaluation using simulated datasets

In order to validate our methods, we have designed a simulated metagenomic study and compared the results with three previous approaches: (i) identifying significantly active subnetworks using simulated annealing and greedy search [13]; (ii) discovering significant individual reactions using Metastats [9]; and (iii) finding differentially abundant KEGG defined pathways, an approach widely used in metagenomic functional comparison [3,7,10]. We choose these tools because they are addressing similar biological problems. However they do not solve the exact same problem as this paper, which is finding differentially abundant subnetworks that may span two or more KEGG defined pathways (see discussion in the Background section). Here the goal of this simulated study is to show that the computational problem in this paper can not be directly solved by applying methods previously developed in a related context.

We designed a simulated metabolic pathways dataset in which five subjects are created for each of the two groups with distinct phenotypes. To generate the artificial reaction abundance matrix (where rows represent reactions and columns represent subjects), a Gaussian distribution is created for each reaction, whose mean is randomly chosen from a real metagenomic dataset (gut microbiome from obese and lean subjects [3]). The variance of each distribution is calculated by setting the relative standard deviation (standard deviation divided by the mean) to 0.2. If we define a reaction to be equally abundant between two groups under comparison, then a random abundance value is generated from the same distribution for each subject. Otherwise, if a reaction is defined to be significantly enriched in one group, then another normal distribution is created for this reaction by increasing the mean such that the *p* value of the difference for the two distributions is less than a predefined value (0.05 and 0.01 were used). In this study, we have chosen a subnetwork (a series of reactions with length 5 or 10) to be enriched in one population. The goal is to compare different methods in recovering this significant subnetwork (a set of significant reactions) based on the simulated abundance matrix. Biologically, the enriched pathways indicate functional enrichment of certain biological processes in a microbial community.

**Figure 3:**
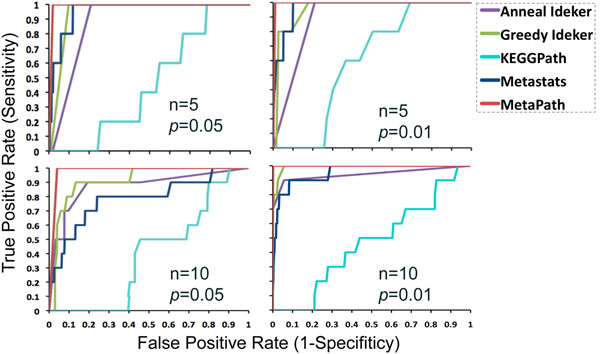
**Comparison of statistical methods for discovering significant reactions in simulated datasets**. Four methods are evaluated: discovering active subnetworks using simulated annealing (Anneal) and greedy search (Greedy) [13], discovering significant individual reactions using Metastats [9], finding differentially abundant KEGGdefined pathways (KEGGPath), and MetaPath. Four datasets are created by varying the number of significant reactions *n* and their significances.

The receiver operating characteristic (ROC) curve is plotted for each method (Fig. 3). Fig. 3 shows that MetaPath outperforms all other methods dramatically showing the advantage in finding significant subnetworks. Note that the results tested on our simulated datasets can be considered as the baseline performance, because it contains only one significant subnetwork, whereas real metagenomic datasets typically contain multiple significant pathways. The most commonly used approach — comparing KEGG-defined pathways — performs the worst in our simulation study (Fig. 3).

### Obese and lean twins

**Figure 4:**
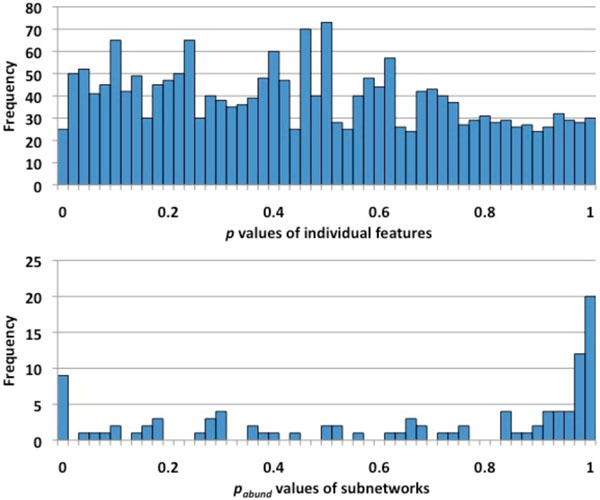
***p* values distributions from comparing individual metabolic reactions by Metastats and from comparing metabolic networks by MetaPath**. The top histogram is the distribution of the p values of individual metabolic reactions calculated by Metastats. The Bottom histogram is the distribution of the p_abund_ values of the subnetworks calculated by MetaPath.

We used MetaPath to compare the abundances of the metabolic networks of the gut microbiome in lean and obese subjects, relying on data from [3]. This metagenomic dataset comprises 6 samples from obese subjects and 6 samples from lean objects. The sequences are annotated and mapped to KEGG reactions using BLASTX (E value < 10^-5^, bitscore > 50, and %identity > 50; parameters suggested in the original study), resulting in total 1832 unique reactions within the 12 metagenomic samples. First, we computed *p* values [16] using Metastats to find differentially abundant reactions. Using a *p* value cutoff of 0.05, 92.7±9.1 (mean±standard deviation) reactions are significant including 37.1±6.6 and 55.6±3.1 enriched reactions in obese and lean groups, respectively, based on 10 runs of Metastats. The high variance of the number of significant genes can be primarily explained by two reasons: (1) some reactions are slightly below or above significance cutoff (0.05), thus *p* values computed through bootstrapping will jump between being considered significant and not significant (Fig. 4); (2) there are large variances of the abundance values within individuals in a same phenotypic group. In addition to *p* values, Metastats also provides an estimate of the False Discovery Rate (*q* value), information that is not used by MetaPath. The *q* values for all reactions are 1 (except R01676 where *q*=0.73), i.e. a literal interpretation of Metastats results would indicate no pathways are significantly different between the two populations. This result can be explained by the flat distribution of the *p* values (Fig. 4), from which the *q* values are estimated. This observation highlights the limitation of relying on the false discovery rate, which requires the estimation of the proportion of features that are truly null [16], approach that does not perform well when only few features are truly significant.

**Figure 5:**
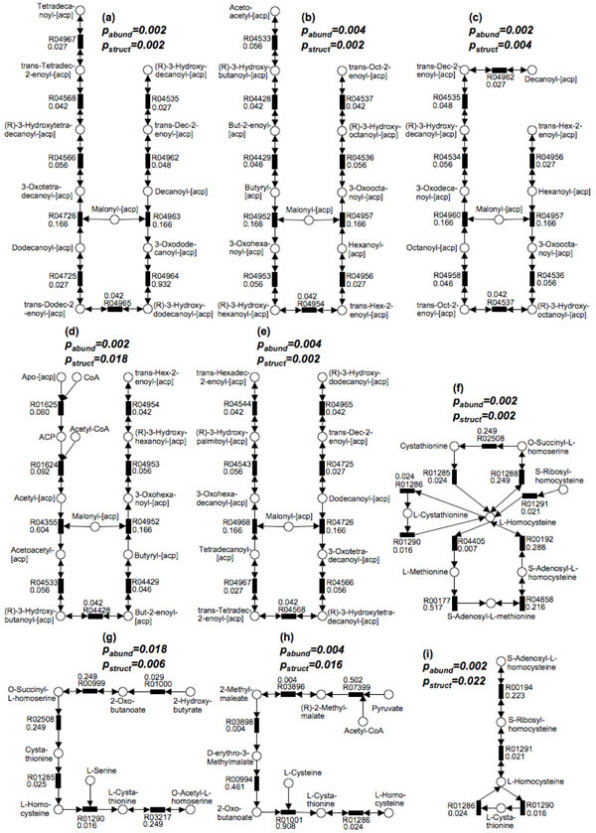
**9 statistically significant subnetworks are found in the comparison of the gut microbiome from the obese and lean subjects**. All these subnetworks are enriched in the obese subjects. p_abund_ and p_struct_ significance values are shown above each subnetwork. p values for each reaction are shown with the KEGG reaction number. Five pathways (a)-(e) belong to the Fatty Acid Metabolism pathway in KEGG. Four pathways (f)-(i) contain the L-Homocysteine molecules.

We, then, applied MetaPath to this dataset, and have found 9 differentially abundant subnetwork (Fig. 5) using 0.05 cutoff value for both *p_abund_* and *p_struct_*. All these subnetworks are enriched in obese subjects; none was found to be enriched in lean subjects. These 9 significant subnetworks contain 48 unique reactions, 22 of which are significant. It is worth pointing out that the number of significant reactions varies between different runs of statistical permutations (using Metastats) as shown above, but the significant pathways identified by Metapath stay the same (Fig. 5). This observation confirms that the results from MetaPath are more robust in the presence of noise in the data than the gene-by-gene approach. In the *p* values distribution of subnetworks (Fig. 4), most of them are either very significant or insignificant and very few are around the *p* value cutoff, allowing the users to easily interpret the results.

Five subnetworks (Fig. 5a-5e) are completely contained in the KEGG Fatty Acid Biosynthesis pathway, which consists of catabolic processes that can generate energy and primary metabolites from fatty acids. Our findings are consistent with previous observations and biochemical analysis in microbiota transplantation experiments in germ-free mice [17], where the concentrations of short-chain fatty acids in the caeca of obese mice are higher than lean mice, suggesting that the gut microbiome in obese subjects has an increased capacity for dietary energy harvest.

Another interesting significant networks consists of 10 reactions (Fig. 5f), of which 8 belong to Cysteine and Methionine Metabolism and 2 belong to Sulfur Metabolism. Many reactions in this subnetwork are connected by the L-Homocysteine molecule. In addition, three other subnetworks (Fig. 5g-5i) we discovered further confirm its potential involvement in obesity, because all these three pathways contain L-homocysteine as metabolite. It is well-known that a high level of blood serum homocysteine is a risk factor for cardiovascular disease [18], and obesity — an increasingly prevalent metabolic disorder — is closely associated with heart disease [19]. Significant correlations between plasma homocysteine concentrations and obesity have been previously reported [18,20-23]. The finding of increased potential for homocysteine metabolism within the obese gut microbiome provides an interesting hypothesis for future studies that, the gut microbiome may either have a direct role in the elevation of homocysteine levels in plasma, or may indirectly affect the hepatic biosynthesis of this amino-acid in the human body.

### Infant and adult individuals

A second data-set comprises gut microbiome samples from 4 infants and 9 adults individuals which were sequenced by Kurokawa, *et al*., 2007 [7]. The sequences were annotated and mapped to the reactions of KEGG pathway using BLASTX (E value < 10^-8^, hit length coverage ≥ 50% of a query sequence), resulting in total 1781 unique reactions within the 13 metagenomic samples. Based on 10 runs of Metastats, 383.7±1.56 reactions are significant using *p* value cutoff of 0.05, including 268.7±1.56 and 115±0 reactions enriched in infant and adult subjects respectively.Using a *q* value cutoff of 0.05, 167.2±2.7 reactions are significant, including 133.2±2.7 and 34±0 reactions enriched in infant and adult subjects respectively.Compared with the previous dataset (obese and lean twins samples), the predictions of significant reactions are much more consistent across different permutations.

**Figure 6:**
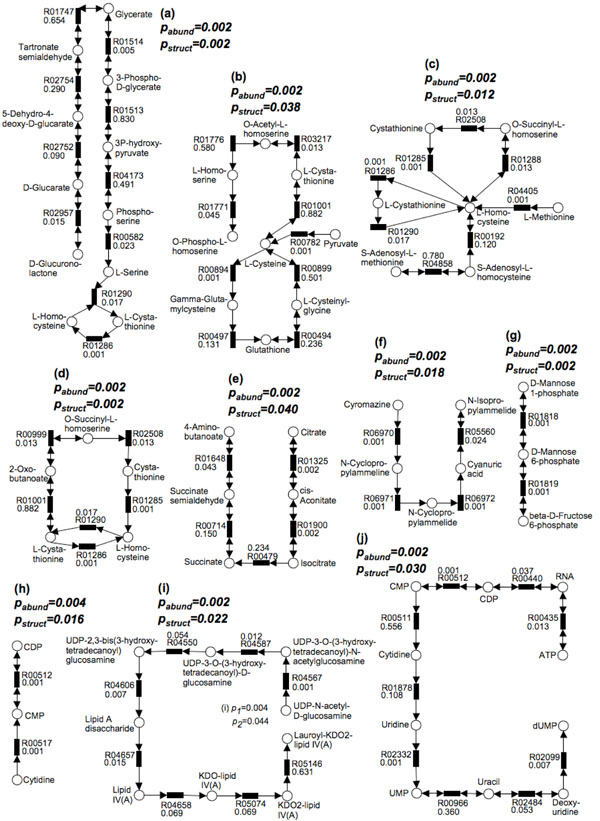
**10 statistically significant subpathways are found in the infant and adult individuals dataset**. 6 subpathways are enriched in the infant subjects (Fig. 4a-4f), and 4 subpathways are enriched in the adult subjects (Fig. 4g-4j). p_abund_ and p_struct_ significance values are shown above each pathway. *p* values for each reaction are shown with the KEGG reaction number.

Applying MetaPath to search for significant subnetworks using the same parameters as before, we have found that 6 are enriched in infant subjects (Fig. 6a-6f) and 4 are enriched in adult subjects (Fig. 6g-6j). These 10 significant subnetworks contain 55 unique reactions (35 and 20 in subnetworks enriched in infant and adult, respectively), including 38 significant reactions (22 and 16 enriched in infant and adult, respectively) and 17 reactions not found significant by Metastats. Three subnetworks enriched in infant subjects (Fig. 6a, 6c and 6d) involve the metabolite L-homocysteine, and a fourth one (Fig. 6b) involves L-cysteine – a related amino-acid, which is consistent with previous observation that breastfed babies have an higher plasma homocysteine level possibly caused by suboptimal availability of folate in breast milk [24]. The concentration of folate is negatively correlated with that of homocysteine, as folate is a necessary coenzyme for reactions that metabolize homocysteine. In addition, babies normally have high protein diet, which may also cause the concentration of homocysteine to increase. A second pathway in Fig. 6e involves substrates citrate and succinate, and is closely related with oxidative tricarboxylic acid (TCA) cycle. TCA cycle is part of carbohydrate metabolism and can convert carbohydrates into usable energy in aerobic organisms. Because the adult gut ecosystem is dominated by strict anaerobes, it is reasonable to find this subpathway enriched in infant individuals where the gut microbiota also includes aerobes. This finding is consistent with results obtained by comparing COG functional categories [7]. We also find a subpathway belonging to atrazine metabolism to be enriched in infant subjects (Fig. 6f). Atrazine is one of the most widely used herbicides, and it contaminates water and soil throughout the world. Our finding possibly indicates a side-effect of this contamination.

The pathway in Fig. 6i (enriched in adult subjects) is part of the lipopolysaccharide biosynthesis. Lipopolysaccharides are a building block of the outer membrane of Gram-negative bacteria. The enrichment of pathway Fig. 6i in adult subject may be a result of the fact that Gram-negative bacteria are also enriched in adults. Specifically, Bacteroides, a genus of Gram-negative bacteria, are a major constituent of adult gut microbiome, but not highly prevalent in infants. Fig. 6h and Fig. 6j (enriched in adult) are pathways related with pyrimidine metabolism. The metabolites RNA, cytidine and uridine, which are contained in pyrimidine metabolism, are normally obtained from high RNA food such as organ meats, broccoli, and brewer’s yeast, which are not available to unweaned infants, as they are not present in high abundance in milk. The pathway in Fig. 6g (enriched in adult) is part of fructose and mannose metabolism a pathway related to carbohydrate metabolism. This is also consistent with COG-based analyses indicating that many mono- or disaccharides metabolism genes are enriched in adults [7], explained by the fact that colonic microbiota in adults uses indigestible polysaccharides as resources for energy production and biosynthesis of cellular components.

## Conclusions

We have introduced a statistical method for finding significant metabolic subpathways from metagenomic datasets. Compared with previous methods, results from MetaPath are more robust to noise in the data, and have significantly higher sensitivity and specificity (when tested on simulated datasets). When applied to two publicly available metagenomic data-sets the output of MetaPath is consistent with previous observations and also provides several new insights into the metabolic activity of the gut microbiome. Finally, MetaPath is efficient: a typical metagenomic dataset and the corresponding metabolic network (about 2000 edges) can be analyzed in half an hour on a single processor.

While showing promising results, our methods have several limitations that we plan to address in the near future. First, and foremost, we restrict ourselves to pathways of a fixed length — a restriction necessary for accurately computing the null distribution of pathway scores. This can severely affect our ability to discover long pathways whose abundance differs only slightly, but significantly, between samples. Second, we currently estimate gene abundances by simply counting the number of sequencing reads that map to a certain gene. Such an approach ignores differences in the length of genes, potentially leading to incorrect conclusions. We plan to address this issue by incorporating a recently-published [12] method that can accurately correct for gene-length effects. The software described in this paper is freely-available under an open-source license from http://metapath.cbcb.umd.edu

## Competing interests

The authors declare that they have no competing interests

## Authors' contributions

BL and MP conceived the project, designed the algorithm and wrote the manuscript. BL implemented the algorithm and analyzed the data. Both authors read and approved the final manuscript.
